# Clinical Use of N-terminal Pro-B-Type Natriuretic Peptide (NT-proBNP) in Diagnosing Acute Heart Failure in Emergency Settings: A Systematic Review

**DOI:** 10.7759/cureus.111120

**Published:** 2026-06-18

**Authors:** Ala Bala Babiker Abdalla, Miada Adel Edris Mohamed, Sahar Isameldin Osman Eisa, Rayan Mohamedani, Lobaba Mubarak Saidahmed Ahmed, Ebraheem Hamzeh, Alaa Kamal Mohamed Abdalla

**Affiliations:** 1 Clinical Pathology, University of Khartoum, Khartoum, SDN; 2 Acute Medicine, Altnagelvin Area Hospital, Western Health and Social Care Trust, Londonderry, IRL; 3 Acute Medicine, St. Vincent's Private Hospital, Dublin, IRL; 4 Pathology, University of Gezira, Wad Madani, SDN; 5 General Practice, Shendi University, Shendi, SDN; 6 General Medicine, Medical University of Lodz, lodz, POL

**Keywords:** acute heart failure, diagnostic accuracy, emergency department, nt-probnp, systematic review

## Abstract

Acute heart failure (AHF) is a common emergency department presentation where rapid and accurate diagnosis is challenging. N-terminal pro-B-type natriuretic peptide (NT-proBNP) is a widely used biomarker for AHF, but uncertainty remains regarding optimal cut-offs and performance across patient subgroups. This systematic review evaluates the diagnostic utility of NT-proBNP for AHF in emergency settings. A systematic review was conducted following the Preferred Reporting Items for Systematic Reviews and Meta-Analyses (PRISMA) guidelines using PubMed, Embase, Scopus, and Web of Science (January 2021-April 2026). Eligible studies enrolled adults with suspected AHF in emergency settings, used NT-proBNP as the index test, and reported diagnostic accuracy outcomes. Risk of bias was assessed using the Quality Assessment of Diagnostic Accuracy Studies-2 (QUADAS-2) tool. A narrative synthesis was performed. Eight studies were included. Across prospective emergency department studies, an NT-proBNP threshold of <300 pg/mL demonstrated strong rule-out performance, with reported sensitivities ranging from 96% to approximately 99% and negative predictive values ranging from 94.8% to 95%, depending on the study population and reference standard. Age-adjusted cut-offs (450/900/1800 pg/mL) produced rule-in specificities of 61-87%. Diagnostic accuracy was significantly reduced in patients with atrial fibrillation (AF), congenital heart disease (CHD), and heart failure with preserved ejection fraction (HFpEF). Five studies had low risk of bias, while three had high risk. NT-proBNP is a highly effective rule-out test for AHF using a <300 pg/mL cut-off. Age-adjusted cut-offs improve rule-in specificity. Performance is attenuated in key subgroups, requiring modified interpretation. Despite some methodological limitations, current evidence strongly supports routine NT-proBNP use in emergency department patients with suspected AHF.

## Introduction and background

Acute heart failure (AHF) is one of the leading causes of emergency department visits and hospital admissions worldwide, particularly among older adults and patients with underlying cardiovascular disease [1]. Early and accurate diagnosis of AHF in emergency settings is essential because delayed recognition may result in increased morbidity, prolonged hospitalization, and higher mortality rates [2]. However, diagnosing AHF in patients presenting with acute dyspnea remains clinically challenging, as its symptoms often overlap with other respiratory and cardiovascular conditions such as chronic obstructive pulmonary disease, pneumonia, pulmonary embolism, and asthma [3]. Traditional diagnostic approaches, including clinical examination, chest radiography, and electrocardiography, may lack sufficient sensitivity and specificity when used alone, especially during the early stages of presentation. Consequently, there has been growing interest in the use of circulating cardiac biomarkers to improve the diagnostic accuracy of AHF in emergency care [4].

N-terminal pro-B-type natriuretic peptide (NT-proBNP) has emerged as one of the most widely studied and clinically utilized biomarkers for the diagnosis of heart failure [5]. NT-proBNP is released from ventricular myocardial cells in response to increased wall stress, pressure overload, and ventricular dysfunction. Elevated NT-proBNP levels have been associated with both acute and chronic heart failure, making the biomarker particularly valuable in patients presenting with undifferentiated dyspnea in emergency departments [6]. Several studies have demonstrated that NT-proBNP possesses high sensitivity and negative predictive value (NPV) for ruling out AHF while also assisting clinicians in risk stratification and treatment decision-making [5,7]. Nevertheless, interpretation of NT-proBNP levels may be influenced by various factors, including age, renal dysfunction, obesity, atrial fibrillation (AF), and other comorbidities, which may affect its overall diagnostic performance across different patient populations and clinical settings.

Over the past few years, numerous original studies have evaluated the clinical utility and diagnostic accuracy of NT-proBNP in emergency settings, with varying cut-off values, methodologies, and reported outcomes. Despite the increasing body of evidence, inconsistencies remain regarding the optimal thresholds and the overall applicability of NT-proBNP across diverse emergency populations. Therefore, a comprehensive synthesis of recent evidence is needed to better understand the current role of NT-proBNP in diagnosing AHF in emergency care. This systematic review aims to evaluate and summarize the available evidence regarding the clinical use, diagnostic performance, and practical implications of NT-proBNP in the diagnosis of AHF in emergency settings.

## Review

Methodology

Study Design

This systematic review was conducted in accordance with the Preferred Reporting Items for Systematic Reviews and Meta-Analyses (PRISMA) guidelines [8] to ensure methodological transparency, reproducibility, and comprehensive reporting.

Eligibility Criteria

The eligibility criteria for study selection were established using the Population, Intervention, Comparator, Outcomes, and Study Design (PICOS) framework [9]. The detailed inclusion and exclusion criteria based on each PICOS element are presented in Table 1.

**Table 1:** Eligibility criteria as per the PICOS framework PICOS: Population, Intervention, Comparator, Outcomes, and Study Design; NT-proBNP: N-terminal pro-B-type natriuretic peptide; PPV: positive predictive value; NPV: negative predictive value; AUC: area under the curve

PICOS element	Inclusion criteria	Exclusion criteria
Population (P)	Adults undergoing NT-proBNP testing for suspected acute heart failure, dyspnea, or heart failure evaluation in emergency departments, acute care settings, hospital-based services, outpatient clinics, or linked healthcare databases	Pediatric populations; animal studies; studies unrelated to heart failure evaluation
Intervention (I)	Measurement of NT-proBNP for diagnostic, prognostic, or clinical evaluation of heart failure	Studies not using NT-proBNP as the primary biomarker of interest
Comparator (C)	Clinical diagnosis of heart failure based on physician assessment, echocardiography, imaging findings, guideline-based criteria, adjudicated diagnosis, healthcare-record linkage, or other predefined clinical reference standards	Studies lacking sufficient clinical outcome assessment or reference criteria
Outcomes (O)	Diagnostic performance outcomes (e.g., sensitivity, specificity, PPV, NPV, AUC) and/or clinically relevant prognostic or healthcare utilization outcomes related to NT-proBNP testing	Studies reporting no relevant diagnostic, prognostic, or clinical outcomes
Study Design (S)	Original observational studies, including prospective studies, retrospective studies, cohort studies, diagnostic accuracy studies, and health-economic evaluations	Reviews, editorials, letters, case reports, conference abstracts, and non-original research

The review included only original research articles published in the English language between January 2021 and April 30, 2026. This time restriction was applied to ensure that the evidence reflects the most recent advances in biomarker-based diagnostics, contemporary emergency department protocols, and updated heart failure management guidelines. Given the rapid evolution of NT-proBNP assay techniques and diagnostic thresholds, older studies were considered less representative of current clinical practice. Therefore, limiting inclusion to recent literature improved the relevance, applicability, and timeliness of the findings. Studies such as review articles, editorials, conference abstracts, case reports, letters to the editor, animal studies, pediatric studies, and studies lacking sufficient diagnostic accuracy data were excluded.

Information Sources and Search Strategy

A comprehensive literature search was conducted to identify all relevant studies evaluating the clinical utility of NT-proBNP for diagnosing AHF in emergency settings. Four major electronic databases were searched, including PubMed, Embase, Scopus, and Web of Science. Medical Subject Headings (MeSH) terms, Emtree terms, keywords, and Boolean operators were combined to construct a highly sensitive search strategy. The full search strategy used across databases is presented in Table 2.

**Table 2:** Search strategy for databases

Database	Detailed search strategy	Filters/limitations applied
PubMed	(“NT-proBNP” OR “N-terminal pro-B-type natriuretic peptide” OR “N terminal pro brain natriuretic peptide” OR “NT-pro brain natriuretic peptide”) AND (“heart failure” OR “acute heart failure” OR “acute decompensated heart failure” OR “cardiac failure” OR “left ventricular failure” OR “acute dyspnea” OR “acute breathlessness”) AND (“emergency department” OR “emergency room” OR “emergency service” OR “emergency care” OR “acute care” OR “critical care”) AND (“diagnosis” OR “diagnostic accuracy” OR “sensitivity” OR “specificity” OR “predictive value” OR “ROC curve” OR “AUC”)	English; human studies; 2021-April 2026
Embase	(‘nt pro bnp’ OR ‘nt-probnp’ OR ‘n terminal pro brain natriuretic peptide’ OR ‘bnp precursor peptide’) AND (‘heart failure’ OR ‘acute heart failure’ OR ‘acute decompensated heart failure’ OR ‘left ventricular dysfunction’ OR ‘cardiac decompensation’) AND (‘emergency department’ OR ‘emergency medicine’ OR ‘emergency care’ OR ‘acute care’ OR ‘critical care’) AND (‘diagnosis’ OR ‘diagnostic accuracy’ OR ‘receiver operating characteristic’ OR ‘roc’ OR ‘sensitivity’ OR ‘specificity’)	English; human; 2021-2026
Scopus	TITLE-ABS-KEY (“NT-proBNP” OR “N-terminal pro-BNP” OR “N-terminal pro brain natriuretic peptide”) AND TITLE-ABS-KEY (“acute heart failure” OR “acute decompensated heart failure” OR “cardiac failure” OR “dyspnea” OR “shortness of breath”) AND TITLE-ABS-KEY (“emergency department” OR “emergency care” OR “emergency room” OR “acute care”) AND TITLE-ABS-KEY (“diagnostic accuracy” OR “sensitivity” OR “specificity” OR “AUC” OR “ROC”)	English; 2021-2026
Web of Science	TS=(“NT-proBNP” OR “N-terminal pro-B-type natriuretic peptide” OR “NT proBNP”) AND TS=(“acute heart failure” OR “heart failure” OR “acute decompensated heart failure” OR “cardiac failure” OR “dyspnea”) AND TS=(“emergency department” OR “emergency care” OR “emergency medicine”) AND TS=(“diagnostic accuracy” OR “sensitivity” OR “specificity” OR “ROC curve” OR “AUC”)	English; 2021-2026

Study Selection

All identified records were imported into EndNote X9 (Clarivate, London, United Kingdom) for organization and duplicate removal. Duplicate studies identified across databases were removed electronically and manually verified to ensure accuracy. Following deduplication, two independent reviewers screened the titles and abstracts of retrieved articles according to the predefined eligibility criteria. Studies considered potentially relevant underwent full-text assessment for final inclusion. Any disagreements during the selection process were resolved through discussion and consensus among the reviewers to minimize selection bias.

*Data Extraction * 

Data extraction was performed independently by the reviewers using a standardized data extraction form developed specifically for this review. Extracted information included study characteristics such as author name, publication year, country, study design, clinical setting, sample size, patient population, NT-proBNP assay methods, cut-off values, reference standards for AHF diagnosis, and key diagnostic performance outcomes including sensitivity, specificity, positive predictive value (PPV), NPV, and area under the curve (AUC) values. Additional information regarding the clinical interpretation and applicability of NT-proBNP in emergency settings was also collected.

Risk-of-Bias Assessment

The methodological quality and risk of bias of the included primary diagnostic accuracy studies were assessed using the Quality Assessment of Diagnostic Accuracy Studies-2 (QUADAS-2) tool [10]. This validated instrument evaluates studies across four key domains, including patient selection, index test, reference standard, and flow and timing. Each domain was assessed for potential risk of bias, while the first three domains were additionally evaluated for concerns regarding applicability. Because QUADAS-2 is designed specifically for primary diagnostic accuracy studies, its application was restricted to the five included studies that reported the accuracy of NT-proBNP against a clinical reference standard for AHF. The two decision-analytic economic evaluations and the prognostic admission-outcome study were not appraised with QUADAS-2: the economic models do not generate primary diagnostic data, and the prognostic study used no formal reference standard for AHF. These three studies were therefore excluded from the QUADAS-2 table and instead appraised narratively for relevance and methodological transparency, with the economic evaluations considered against accepted expectations for decision-analytic models (appropriateness and source of input parameters, model structure, and reporting) and the prognostic study treated as supportive rather than diagnostic evidence. Each domain judgement was made independently by two reviewers, with disagreements resolved by consensus, and ratings were downgraded where the reference standard, patient selection, or timing introduced a credible risk of bias.

Data Synthesis

A qualitative narrative synthesis of the included studies was conducted to summarize and compare the findings regarding the diagnostic utility of NT-proBNP in AHF. A meta-analysis was not performed due to substantial clinical and methodological heterogeneity among the included studies. Significant variations were observed in NT-proBNP cut-off values, assay techniques, patient populations, clinical settings, diagnostic reference standards, and reported outcome measures. Additionally, differences in age distributions, comorbid conditions such as renal dysfunction and AF, and inconsistent reporting of diagnostic parameters limited the comparability of the studies. Performing a pooled quantitative analysis under such heterogeneous conditions could have produced misleading or clinically unreliable summary estimates. Therefore, a narrative synthesis was considered the most appropriate approach to ensure the accurate interpretation and contextual evaluation of the available evidence.

Results

Study Selection Process

**Figure 1:**
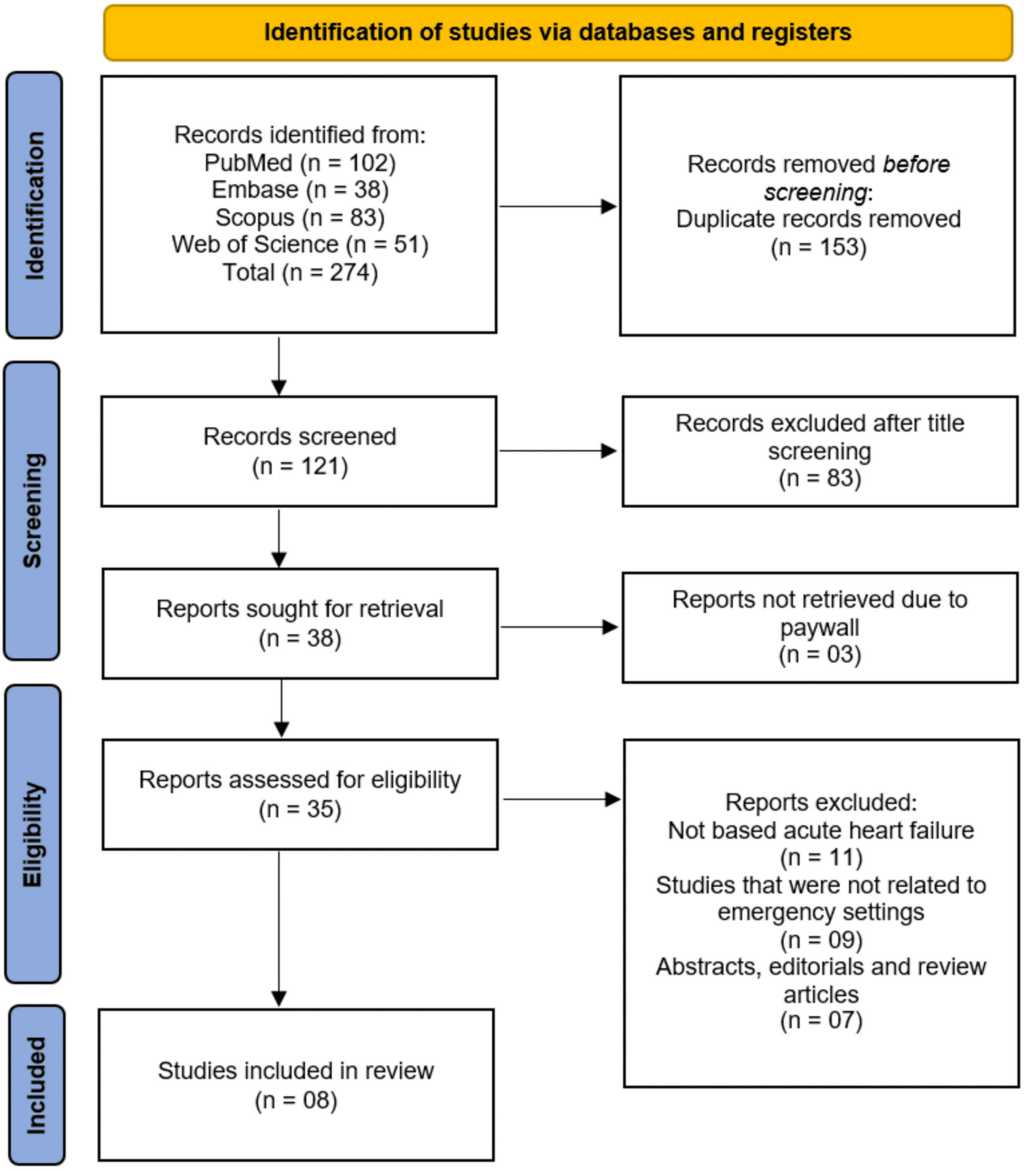
PRISMA flowchart PRISMA: Preferred Reporting Items for Systematic Reviews and Meta-Analyses

The study selection process followed the PRISMA guidelines, as summarized in the flow diagram (Figure 1). A total of 274 records were identified from four electronic databases: PubMed (n=102), Embase (n=38), Scopus (n=83), and Web of Science (n=51). After the removal of duplicate records (n=153), 121 unique records remained for title screening. Following title screening, 83 records were excluded as they were not relevant to the research question, leaving 38 reports sought for retrieval. Of these, three reports could not be retrieved due to paywall restrictions despite database access attempts and were therefore excluded from full-text assessment. This may have introduced selection bias and is acknowledged as a limitation of the review. The remaining 35 reports were assessed for full‑text eligibility. Following a detailed eligibility assessment, 27 reports were excluded for the following reasons: not based on AHF (n=11), studies that were not related to emergency settings (n=9), and abstracts, editorials, or review articles (n=7). Ultimately, eight studies [11-18] met the predefined inclusion criteria and were included in this systematic review.

Key Characteristics of the Studies

A total of eight studies [11-18] were included in this systematic review, all of which evaluated the diagnostic performance or clinical utility of NT‑proBNP for AHF in emergency settings. Their key characteristics are summarized in Table 3. The studies originated from the USA [11,13], the UK [12,15], Switzerland [14], Ireland [17], and a multi‑country economic analysis (UK, the Netherlands, Spain) [16]. Study designs comprised four retrospective cohorts [11,12,15,17], two prospective cohorts [13,14], and two decision‑analytic economic models [16,18]; note that Guidi et al. [13] and Belkin et al. [14] were prospective multicenter trials. Sample sizes varied widely, from 469 patients in a hospital‑based diagnostic study [15] to over 155,000 individuals in a primary care‑hospital linkage study [12]. All studies enrolled adults with suspected AHF or dyspnea in emergency departments or acute medical admission units, except for Birrell et al. [15], who recruited patients from non‑emergency department hospital settings with echocardiography and NT‑proBNP testing. The reference standard for AHF diagnosis was clinical adjudication by expert panels or predefined clinical criteria (symptoms, signs, congestion, diuretic escalation) in all studies except Conway et al. [17], who used International Classification of Diseases (ICD)-coded outcomes without a formal AHF gold standard, and Jones et al. [12], who employed Clinical Practice Research Datalink (CPRD)-Hospital Episode Statistics (HES) linkage with six-month follow-up.

**Table 3:** Characteristics of the included studies ED: emergency department; ER: emergency room; HDU: high-dependency unit; NT-proBNP: N-terminal pro-B-type natriuretic peptide; AHF: acute heart failure; HF: heart failure; CPRD: Clinical Practice Research Datalink; HES: Hospital Episode Statistics; Li-heparin: lithium heparin; CEC: clinical events committee; EF: ejection fraction; BSE: British Society of Echocardiography; AMAU: acute medical assessment unit; ICU: intensive care unit; IQR: interquartile range; ECLIA: electrochemiluminescence immunoassay; ICD: International Classification of Diseases; LOS: length of stay

Study (author, year)	Country	Study design	Setting (ED/ER/ICU)	Sample size	Population (age/criteria)	Index test (NT-proBNP assay)	Reference standard for AHF	Key aim
Ali et al. (2026) [11]	USA	Retrospective cohort	ED + outpatient	4,648	Adults ≥18 years with congenital heart disease (biventricular)	NT-proBNP (pg/mL cut-offs: 125, 250, 300, 450)	Clinical HF diagnosis (symptoms/signs + congestion + diuretic escalation, adjudicated)	Evaluate diagnostic accuracy of NT-proBNP for HF rule-in/rule-out in ED and outpatient settings
Jones et al. (2025) [12]	UK	Retrospective cohort	Primary care + hospital linkage	155,347	≥45 years, NT-proBNP tested (2004-2018), no prior HF	NT-proBNP (125-2000 pg/mL)	HF diagnosis (CPRD + HES, 6-month follow-up)	Assess NT-proBNP accuracy for HF diagnosis in real-world practice
Guidi et al. (2025) [13]	USA	Prospective multicenter trial	ED (9 hospitals)	598 (490 analyzed)	≥21 years, suspected AHF, dyspnea, age <50/50-75/>75	Beckman Access NT-proBNP (Li-heparin)	CEC adjudication (HF vs. non-HF) using full clinical data	Diagnostic accuracy of NT-proBNP for AHF in ED + age-based cut-offs and assay comparison
Belkin et al. (2025) [14]	Switzerland	Prospective cohort (diagnostic/prognostic)	ED (2 university hospitals)	1,400	Adults with acute dyspnea; median age 76 years; AHF suspected	NT-proBNP (Elecsys and Access assays)	Expert adjudication of AHF using full clinical data	Assess diagnostic accuracy, agreement, and prognosis of NT-proBNP for AHF in ED dyspnea patients
Birrell et al. (2024) [15]	UK (Scotland)	Retrospective cohort	Hospital (non-ED, lab + echo)	469	Suspected HF, 26-94 years; selected echo + NT-proBNP cases; excluded AF, low EF, valve disease	NT-proBNP (125-2000 ng/L cut-offs)	Echocardiography (BSE diastolic grading)	Assess diagnostic value of NT-proBNP vs. diastolic dysfunction
Walkley et al. (2023) [16]	UK (also Netherlands and Spain scenarios)	Economic model (decision tree + Markov)	ED	1000	Adults with suspected AHF	NT-proBNP rule-in/rule-out (±300 pg/mL rule-in/rule-out)	Clinical diagnosis pathway (ICON-RELOADED, BASEL V data)	Cost-effectiveness of NT-proBNP strategies in ED AHF diagnosis
Conway et al. (2023) [17]	Ireland	Retrospective cohort	ED → AMAU (including ICU/HDU)	64,212 admissions	Adults; median age 64 years (IQR: 45–78)	NT-proBNP (Roche Elecsys ECLIA)	No formal AHF gold standard; ICD + outcomes	Assess NT-proBNP vs. mortality, LOS, utilization
Siebert et al. (2021) [18]	USA	Decision model	ED	1,424	Dyspneic adults	NT-proBNP (rule-in/rule-out)	ICON-RELOADED dx	Compare NT-proBNP vs. clinical dx cost/outcomes

Diagnostic Performance of NT‑proBNP for AHF

Table 4 presents the diagnostic accuracy metrics for NT‑proBNP across the included studies [11-18]. Overall, NT‑proBNP demonstrated high sensitivity for rule‑out and moderate to high specificity for rule‑in of AHF, though performance varied by cut‑off value, age, and comorbidity status.

**Table 4:** Diagnostic performance of NT-proBNP for AHF PPV: positive predictive value; NPV: negative predictive value; AUC: area under the curve; ROC: receiver operating characteristic; OP: outpatient; ED: emergency department; NT-proBNP: N-terminal pro-B-type natriuretic peptide; AHF: acute heart failure; NR: not reported

Study (author, year)	Cut-off value (pg/mL)	Sensitivity (%)	Specificity (%)	PPV (%)	NPV (%)	AUC (ROC)	Clinical interpretation
Ali et al. (2026) [11]	<125 (OP)/<300 (ED); ≥250 (OP)/≥450 (ED)	86/82/74/58	59/53/68/75	87/36/76/88	98/91/71/36	0.667/0.648/0.699/0.657	OP: rule-out/rule-in; ED: rule-out/rule-in
Jones et al. (2025) [12]	125/400/660	98.8-77.1	13.2-84.9	14-26	94-99	0.743-0.877	High NPV, good rule-out test; accuracy ↓ in AF, ↑ false positives at low cut-offs
Guidi et al. (2025) [13]	<300/450/900/1800	96/84-90/87-90	-/81-70-61	72-74	95-93-79	0.87	<300 rule-out; age-cuts rule-in (↓spec with age)
Belkin et al. (2025) [14]	<300 (rule-out); age-adjusted rule-in (450-1800)	~98.5-98.9	~77.6-84.8	NR	High	0.914-0.922	Excellent rule-out; good rule-in accuracy
Birrell et al. (2024) [15]	125/400/1000/2000	83.4/43.3/18.9/9.2	25.0/67.9/88.0/96.4	52/57/60/71	61/55/53/52	NR	Low cut-off = rule-out; high cut-offs = rule-in ↑ specificity
Walkley et al. (2023) [16]	300 (rule-in/rule-out strategy)	95.3	66.2	68.4	94.8	NR	High sensitivity + moderate specificity; useful ED rule-in/rule-out; reduces admissions while maintaining safety
Conway et al. (2023) [17]	<50/≥100/≥250/≥1000/≥3000	NR	NR	NR	NR	NR	NT-proBNP stratified into risk groups only; no diagnostic accuracy reported; higher levels linked with increasing mortality risk
Siebert et al. (2021) [18]	<300 (rule-out); >450/900/1800 (rule-in by age)	79/94	87/72	NR	NR	NR	Dual cut-off NT-proBNP: high rule-out (94%) + good rule-in (87%); grey zone = clinical assessment

Using a rule‑out cut‑off of <300 pg/mL, three large emergency department‑based studies reported consistently high sensitivities of 95-99% and NPVs of 94-99% [13,14,16]. Belkin et al. [14] found that the <300 pg/mL threshold achieved a sensitivity of approximately 98.5-98.9% and a specificity of 77.6-84.8% using two different NT‑proBNP assays (Elecsys and Access), with excellent AUCs of 0.914-0.922. Similarly, Walkley et al. [16] reported a sensitivity of 95.3% and specificity of 66.2% for the 300 pg/mL rule‑in/rule‑out strategy, resulting in a high NPV of 94.8%. Guidi et al. [13] confirmed that <300 pg/mL was effective for rule‑out (sensitivity: 96%) and age‑specific rule‑in cut‑offs (450, 900, and 1800 pg/mL for age <50, 50-75, and >75 years) yielded specificities of 81%, 70%, and 61%, respectively, with an overall AUC of 0.87.

In the retrospective analysis by Ali et al. [11] among adults with congenital heart disease, the emergency department rule‑in cut‑off ≥450 pg/mL had a sensitivity of 58% and a specificity of 75% (PPV: 36%; NPV: 91%), while the outpatient rule‑out cut‑off <125 pg/mL showed 86% sensitivity and 59% specificity. AUCs ranged from 0.648 to 0.699, which were lower than in general AHF populations, suggesting reduced accuracy in this specific subgroup. Jones et al. [12] evaluated NT‑proBNP in patients with AF and found that accuracy declined substantially in AF compared to non‑AF patients; for a cut‑off of 125 pg/mL, sensitivity was 98.8% but specificity only 13.2%, whereas increasing the cut‑off to 400 pg/mL and 660 pg/mL improved specificity to 67.9% and 84.9%, respectively, albeit with lower sensitivity. The AUC ranged from 0.743 to 0.877, and the authors concluded that NT‑proBNP remains a useful rule‑out test in AF when lower cut‑offs are avoided.

In a study focusing on heart failure with preserved ejection fraction (HFpEF), Birrell et al. [15] reported that a low cut‑off of 125 pg/mL gave 83.4% sensitivity but only 25% specificity, whereas higher cut‑offs (1000 and 2000 pg/mL) progressively increased specificity to 88% and 96.4% at the expense of sensitivity (18.9% and 9.2%, respectively). No AUC was reported, but the authors noted that NT‑proBNP was useful for ruling out HFpEF at low thresholds and ruling it in at high thresholds.

Siebert et al. [18] evaluated a dual cut‑off strategy in an economic decision model: <300 pg/mL for rule‑out (sensitivity: 94%; specificity: 72%) and age‑adjusted rule‑in values (>450/>900/>1800 pg/mL) with a sensitivity of 79% and a specificity of 87%. The intermediate "grey zone" mandated clinical assessment. Conway et al. [17] did not report traditional diagnostic accuracy metrics; instead, they stratified NT‑proBNP into risk categories (<50, ≥100, ≥250, ≥1000, ≥3000 pg/mL) and found progressively higher in‑hospital mortality with increasing levels, but no formal sensitivity or specificity for AHF diagnosis was provided.

Summary of Key Findings Across the Studies

The studies most directly addressing the diagnosis of AHF in emergency department patients were the prospective multicenter investigations by Guidi et al. [13] and Belkin et al. [14], both of which evaluated NT-proBNP in adults presenting with suspected AHF or acute dyspnea and demonstrated excellent rule-out performance for a <300 pg/mL threshold. Guidi et al. [13] reported a sensitivity of 96%, and Belkin et al. [14] reported sensitivities of approximately 98.5-98.9%, supporting the utility of this threshold for excluding AHF in emergency settings. Age-adjusted rule-in cut-offs (450/900/1800 pg/mL) improved specificity and were supported by these studies as well as by Siebert et al. [18].

The remaining studies provided complementary evidence but differed from the primary review population. Ali et al. [11] evaluated adults with congenital heart disease in both emergency and outpatient settings, Jones et al. [12] used a primary care-hospital linkage cohort that included patients undergoing NT-proBNP testing for heart failure evaluation, and Birrell et al. [15] assessed NT-proBNP in a non-emergency hospital population with suspected HFpEF. Conway et al. [17] reported prognostic rather than diagnostic outcomes, while Walkley et al. [16] and Siebert et al. [18] were economic evaluations informed by existing diagnostic data rather than primary diagnostic accuracy studies.

Overall, the evidence supports the diagnostic value of NT-proBNP for AHF rule-out in emergency settings, particularly from prospective emergency department-based studies. However, the inclusion of non-emergency department populations, disease-specific cohorts, administrative datasets, and economic models introduces heterogeneity and should be considered when interpreting the applicability of the findings to routine emergency department practice.

Risk-of-Bias Assessment

The risk of bias of the five primary diagnostic accuracy studies [11-15] was assessed using the QUADAS-2 tool, and the results are presented in Table 5. Consistent with the intended scope of QUADAS-2, the two decision-analytic economic evaluations [16,18] and the prognostic admission-outcome study [17] were not appraised with this tool and have been removed from Table 3; they are considered separately below. Following the re-appraisal of each domain by two reviewers, two of the five diagnostic studies were judged to be at low overall risk of bias, one at unclear risk, and two at high risk.

**Table 5:** Risk-of-bias assessment using QUADAS-2 QUADAS-2: Quality Assessment of Diagnostic Accuracy Studies-2; NT-proBNP: N-terminal pro-B-type natriuretic peptide

Study (author, year)	Patient selection	Index test (NT‑proBNP)	Reference standard	Flow and timing	Overall risk of bias
Ali et al. (2026) [11]	Unclear	Low	Low	Unclear	Unclear
Jones et al. (2025) [12]	Low	Low	High	High	High
Guidi et al. (2025) [13]	Low	Low	Low	Low	Low
Belkin et al. (2025) [14]	Low	Low	Low	Low	Low
Birrell et al. (2024) [15]	High	Unclear	Unclear	Unclear	High

Guidi et al. [13] and Belkin et al. [14] were rated at low risk across all four domains. Both were prospective multicenter studies that enrolled patients with suspected AHF, applied pre-specified NT-proBNP thresholds, and used blinded expert or clinical events committee adjudication of the final diagnosis based on complete clinical data, with the index test and reference standard performed contemporaneously. These two studies therefore provide the most methodologically secure diagnostic evidence in this review.

Ali et al. [11] was downgraded from low to unclear overall risk. The reference standard (adjudicated clinical heart failure diagnosis) and the pre-specified index test thresholds were judged at low risk; however, the patient selection domain was rated unclear because the study was retrospective and confined to a selected congenital heart disease population, so it is uncertain whether enrolment was consecutive and representative, and the flow-and-timing domain remained unclear because the interval between testing and reference standard ascertainment was not fully reported. This study also carries a high applicability concern for the general undifferentiated emergency population.

Jones et al. [12] was downgraded from low to high overall risk. Patient selection and the index test were judged at low risk, but the reference standard was rated high risk because heart failure status was defined from routinely collected administrative data (CPRD primary care and HES hospital coding) rather than contemporaneous expert adjudication, which is a recognized source of outcome misclassification. The flow-and-timing domain was also rated high risk because the diagnosis was ascertained over a six-month follow-up window after the index test, allowing incident (post-test) heart failure to be counted against a baseline measurement and introducing a clear reference standard and timing concern. The diagnostic estimates from this study should therefore be interpreted with caution.

Birrell et al. [15] remained at high overall risk. Patient selection was rated high risk because of the exclusion of key subgroups (AF, low ejection fraction, and valve disease) and recruitment from a non-emergency hospital setting, which limits representativeness; the index test and flow-and-timing domains were unclear; and the reference standard was downgraded from low to unclear because echocardiographic diastolic grading is an imperfect proxy for clinically adjudicated AHF and blinding of its interpretation was not described.

The two economic evaluations [16,18] and the prognostic study [17] were appraised narratively rather than with QUADAS-2. The decision-analytic models of Walkley et al. [16] and Siebert et al. [18] were reasonably reported but derived their diagnostic accuracy inputs from external literature rather than from primary patient data and therefore cannot independently corroborate the diagnostic performance of NT-proBNP. Conway et al. [17] reported no diagnostic accuracy metrics and used no formal reference standard for AHF, relying instead on ICD coding and clinical outcomes, and is best regarded as prognostic rather than diagnostic evidence.

Carrying this re-appraisal through to the strength of the evidence, the excellent rule-out performance of the <300 pg/mL threshold now rests principally on the two low-risk prospective studies [13,14], because the remaining supporting studies either were reclassified as high risk [12] or fall outside the diagnostic appraisal as economic models [16,18]. Accordingly, the conclusions of this review have been tempered, and the diagnostic accuracy estimates derived from the unclear- and high-risk studies [11,12,15] should be interpreted with corresponding caution.

Discussion

This systematic review synthesized evidence from eight studies evaluating the clinical use of NT-proBNP for diagnosing AHF in emergency settings. The findings indicate that NT-proBNP is a useful biomarker for ruling out AHF when a single cut-off of <300 pg/mL is applied and that age-adjusted cut-offs improve rule-in specificity. However, diagnostic performance varies significantly according to patient subgroups, including those with AF, congenital heart disease, and HFpEF. Importantly, the strength of these conclusions is constrained by the nature of the evidence base: of the eight studies, only five provided primary diagnostic accuracy data and two of these were at low risk of bias, while two were economic decision models and one reported no diagnostic accuracy, and the reference standards differed markedly across studies. These features, rather than sitting only in the limitations, shaped how the findings were grouped and weighted below.

Among the included studies, the most consistent diagnostic finding was the excellent rule-out performance of an NT-proBNP threshold of <300 pg/mL, with sensitivities of 95-99% and NPVs of 94-99% reported by the two prospective emergency department cohorts and reproduced in the two economic decision models [13,14,16,18]. Crucially, however, only the prospective cohorts generated primary patient-level accuracy data; the two decision-analytic models derived their sensitivity and predictive values from earlier trials (ICON-RELOADED and BASEL) rather than from their own patients, so their estimates are not independent diagnostic evidence and are considered here only in the cost-effectiveness context discussed below. This primary data finding is nonetheless consistent with external background literature, such as the PRIDE study, which reported a sensitivity of 99% for excluding AHF at a 300 pg/mL cut-off in dyspneic emergency department patients [19]. Restricted to the studies that provide primary diagnostic data, this rule-out estimate rests on the two prospective trials [13,14]; the two economic models [16,18] reuse the same external estimates and therefore cannot independently corroborate it, though they inform the cost-effectiveness analysis discussed below. From a clinical perspective, the high NPV means that an emergency department physician can be reasonably confident that a patient with dyspnea and an NT-proBNP level below 300 pg/mL is unlikely to have AHF, thereby reducing unnecessary admission or further cardiac imaging.

For the rule-in of AHF, the picture is more nuanced. Age-adjusted cut-offs (450 pg/mL for age <50 years, 900 pg/mL for ages 50-75 years, and 1800 pg/mL for age >75 years) were evaluated by two prospective primary studies and one economic decision model [13,14,18] and produced specificities ranging from 61% to 87%, with correspondingly lower sensitivities; only the two prospective studies, however, contribute primary diagnostic data to this estimate. The modest PPVs reported in some studies [11,12] indicate that a positive NT‑proBNP result should not be interpreted in isolation; rather, it should prompt the consideration of alternative causes of elevated natriuretic peptides, such as renal impairment, pulmonary hypertension, or sepsis, all of which are common in the emergency department population.

Subgroup analyses revealed important limitations in specific patient populations. In patients with AF, Jones et al. [12] demonstrated that the standard low cut-offs produce unacceptably low specificity (13% at 125 pg/mL) due to chronically elevated NT-proBNP levels. This finding from an included study is consistent with external background literature; Knudsen et al. previously showed that patients with AF have NT-proBNP levels two- to three-fold higher than those in sinus rhythm, even in the absence of heart failure [20]. Consequently, higher rule‑in cut‑offs (e.g., 400-660 pg/mL) are required in AF patients to maintain diagnostic specificity, although this reduces sensitivity. Similarly, Ali et al. [11] found lower diagnostic accuracy (AUCs: 0.648-0.699) in adults with congenital heart disease, a population in which baseline NT‑proBNP levels are often elevated due to ventricular pressure or volume overload. These findings highlight that a single algorithm cannot be applied universally and clinicians must interpret NT‑proBNP results in the context of each patient's baseline characteristics.

In the specific subset of HFpEF, Birrell et al. [15] showed that low cut-offs (125 pg/mL) provide good sensitivity (83%) but very poor specificity (25%), whereas high cut-offs (1000-2000 pg/mL) achieve high specificity (88-96%) but miss most cases; this finding derives from a single high risk-of-bias study and should be weighted accordingly. It is broadly consistent with the external Heart Failure Association Pretest Assessment, Echocardiography, Functional Testing, and Final Etiology (HFA-PEFF) and Heavy, Hypertensive, Atrial Fibrillation, Pulmonary Hypertension, Elderly, and Filling Pressures (H2FPEF) diagnostic algorithms, which incorporate NT-proBNP as a major criterion but require higher thresholds (e.g., >220 pg/mL for sinus rhythm and >660 pg/mL for AF) for diagnosing HFpEF [21]. The difficulty in ruling in HFpEF with NT‑proBNP alone reflects the fact that many patients with dyspnea and preserved ejection fraction have alternative diagnoses such as chronic obstructive pulmonary disease, deconditioning, or obesity, all of which can elevate NT‑proBNP to a lesser degree.

Two studies in this review [16,18] were economic decision models rather than primary diagnostic accuracy studies and are therefore discussed here only in terms of cost-effectiveness rather than as sources of diagnostic accuracy data. Both concluded that NT-proBNP-supported strategies reduce unnecessary hospital admissions and are cost-effective compared with clinical judgment alone. These cost-effectiveness conclusions are broadly consistent with an external UK-based analysis by Collinson et al. [22], which found that point-of-care BNP testing in the emergency department reduced admissions and saved approximately £1,200 per patient. Importantly, because both Walkley et al. [16] and Siebert et al. [18] derived their diagnostic accuracy inputs from prior trials rather than from their own patients, their reported sensitivity and predictive values were not treated as independent diagnostic evidence in this review, which avoids double-counting the underlying source data. Their contribution is therefore confined to the economic case for NT-proBNP-supported triage, which itself remains contingent on prospective validation.

On re-appraisal, the studies that had initially appeared to be at low overall risk of bias [11-14,16] did not all withstand closer scrutiny: on the re-appraisal reported above, only the two prospective primary studies remained at low risk, whereas one study was downgraded to unclear and another to high risk, and one was an economic model excluded from the diagnostic appraisal. The studies carrying the greatest methodological limitations [15,17] warrant particular caution. Conway et al. [17] was the only study that did not report any diagnostic accuracy metrics and used no formal reference standard for AHF, instead relying on ICD codes and outcomes. This study is better interpreted as a prognostic rather than a diagnostic evaluation, and its inclusion in a diagnostic systematic review is a limitation. Birrell et al. [15] had high risk due to selective patient exclusions (excluding AF, low ejection fraction, and valve disease), which limits generalizability to the typical heterogeneous emergency department population. Consistent with the external methodological literature, a systematic review by Smith et al. [23] also highlighted that excluding key subgroups such as AF and chronic kidney disease is a common source of bias in NT‑proBNP diagnostic studies. Therefore, the findings from these high‑risk studies should be interpreted cautiously, and future diagnostic studies should explicitly report performance in these important subgroups.

Another important consideration is the variation in NT‑proBNP assays across studies. Belkin et al. [14] directly compared the Elecsys and Access assays and found excellent agreement, with nearly identical diagnostic performance (AUCs: 0.914 vs. 0.922). This included-study finding suggests that assay-related variability is unlikely to be a major concern in clinical practice and is supported by external evidence from a large international harmonization study that demonstrated acceptable inter-assay comparability for NT-proBNP, unlike BNP, which shows greater variability [24]. Guidi et al. [13] also evaluated a novel Beckman Access assay and reported performance consistent with established assays, further supporting generalizability.

It is worth noting that none of the included studies specifically evaluated the utility of serial NT-proBNP measurements in the emergency department nor did they assess the incremental value of NT-proBNP when added to clinical decision rules such as the HEART score or the Ottawa Heart Failure Risk Scale. Outside the included studies, a recent study by Miró et al. [25] found that combining NT‑proBNP with the Ottawa Rule improved specificity for AHF diagnosis compared with either test alone. This represents an important gap for future research.

Limitations

This systematic review has several limitations. First, the body of primary diagnostic evidence is small and of uneven quality: only five of the eight included studies provided primary diagnostic accuracy data, and only two of these were at low risk of bias on QUADAS-2, with the studies carrying the most serious methodological limitations [15,17] and one further primary study rated at unclear risk [11]; therefore, the overall quality of the evidence is not uniformly high. Second, there was substantial heterogeneity in study designs, including retrospective cohorts, prospective trials, and economic models, which limits the ability to pool results quantitatively or perform a meta-analysis. Third, and most consequentially for interpretation, the reference standard for AHF differed so markedly across studies that they cannot be regarded as estimating the same target condition: some used contemporaneous expert adjudication [13,14], others administrative or ICD code linkage [12,17], and one echocardiographic diastolic grading [15]. Because these reference standards capture different constructs, their accuracy estimates were not pooled or treated as interchangeable; instead, this heterogeneity guided how studies were grouped and weighted in the synthesis and places a firm ceiling on the strength of the conclusions that can be drawn. Fourth, the review excluded non-English language studies and did not search grey literature, raising the possibility of publication bias. Fifth, none of the included studies were conducted in low- or middle-income countries, so the generalizability of findings to resource-limited settings is uncertain. Sixth, several studies did not report key diagnostic accuracy metrics such as PPV or likelihood ratios [14,17,18], which are clinically useful for bedside decision‑making. Finally, publication bias cannot be excluded, as studies reporting favorable diagnostic performance may be more likely to be published.

## Conclusions

NT-proBNP appears to be a useful diagnostic adjunct for AHF in emergency settings. A rule-out threshold of <300 pg/mL showed high sensitivity and NPV, although this finding rests principally on two prospective primary studies at low risk of bias, with the remaining supporting evidence drawn from studies at higher risk of bias or from economic models that reused external accuracy estimates. Age-adjusted cut-offs may improve rule-in specificity and can assist in confirming AHF. Diagnostic performance is attenuated in key subgroups, including patients with AF, congenital heart disease, and HFpEF, where modified cut-offs or alternative diagnostic approaches are required. The strength of these conclusions is constrained by methodological heterogeneity, a small number of primary diagnostic studies, and reference standards that differed substantially across studies and so did not estimate the same target condition. Future prospective studies should standardize the reference standard, report diagnostic accuracy across relevant subgroups, and evaluate the incremental value of NT-proBNP when combined with clinical decision rules. Within these constraints, the available evidence supports NT-proBNP as a helpful aid to ruling out AHF in emergency department patients with suspected disease, rather than as a stand-alone test, and its routine adoption should be guided by the higher-quality primary studies and confirmed in further prospective research.
